# Identification of gut metabolites associated with Parkinson’s disease using bioinformatic analyses

**DOI:** 10.3389/fnagi.2022.927625

**Published:** 2022-07-26

**Authors:** Jun Yan, Xia Feng, Xia Zhou, Mengjie Zhao, Hong Xiao, Rui Li, Hong Shen

**Affiliations:** ^1^Department of Geriatric, Nanjing Medical University Affiliated Brain Hospital, Nanjing, China; ^2^Department of Pharmacy, Nanjing Medical University Affiliated Brain Hospital, Nanjing, China; ^3^Institute of Neuropsychiatry, Nanjing Medical University Affiliated Brain Hospital, Nanjing, China; ^4^School of Pharmacy, Nanjing Medical University, Nanjing, China

**Keywords:** gut metabolites, environmental exposures, Comparative Toxicogenomics Database, visual analyses, Parkinson’s disease

## Abstract

**Background:**

Parkinson’s disease (PD) is a common neurodegenerative disease affecting the movement of elderly patients. Environmental exposures are the risk factors for PD; however, gut environmental risk factors for PD are critically understudied. The proof-of-concept study is to identify gut metabolites in feces, as environmental exposure risk factors, that are associated with PD and potentially increase the risk for PD by using leverage of known toxicology results.

**Materials and methods:**

We collected the data regarding the gut metabolites whose levels were significantly changed in the feces of patients with PD from the original clinical studies after searching the following databases: EBM Reviews, PubMed, Embase, MEDLINE, and Elsevier ClinicalKey. We further searched each candidate metabolite-interacting PD gene set by using the public Comparative Toxicogenomics Database (CTD), identified and validated gut metabolites associated with PD, and determined gut metabolites affecting specific biological functions and cellular pathways involved in PD by using PANTHER tools.

**Results:**

Sixteen metabolites were identified and divided into the following main categories according to their structures and biological functions: alcohols (ethanol), amino acids (leucine, phenylalanine, pyroglutamic acid, glutamate, and tyrosine), short-chain fatty acids (propionate and butyrate), unsaturated fatty acids (linoleic acid and oleic acid), energy metabolism (lactate, pyruvate, and fumarate), vitamins (nicotinic acid and pantothenic acid), and choline metabolism (choline). Finally, a total of three identified metabolites, including butyrate, tyrosine, and phenylalanine, were validated that were associated with PD.

**Conclusion:**

Our findings identified the gut metabolites that were highly enriched for PD genes and potentially increase the risk of developing PD. The identification of gut metabolite exposures can provide biomarkers for disease identification, facilitate an understanding of the relationship between gut metabolite exposures and response, and present an opportunity for PD prevention and therapies.

## Introduction

Parkinson’s disease (PD) is a common neurodegenerative disease that is known to affect the movement in the elderly patients and is mainly characterized by the classical motor syndromes, such as resting tremor, rigidity, bradykinesia, akinesia, and postural instability, and accompanied by early non-motor symptoms (NMSs; Balestrino and Schapira, 2020). The pathological hallmarks of PD are the death of dopaminergic neurons in the substantia nigra pars compacta (SNpc) and the presence of Lewy bodies with abnormal aggregates of α-synuclein protein, which leads to dopamine (DA) deficiency within the basal ganglia and movement disorder (Kalia and Lang, 2015). Nevertheless, the exact cause of PD is still poorly understood.

Substantial evidence strongly supports environmental exposures as the risk factor for PD (Noyce et al., 2012). Earlier epidemiological and sociological studies revealed that exposure to toxic environmental substances (including pesticide exposure, prior head injury, and beta-blocker use) and specific living conditions (including rural living, agricultural occupation, and drinking well water) are associated with an increased risk of PD (Langston et al., 1983). However, based on the PD progress, it is unlikely that the disease pathogenesis is triggered by acute toxic exposure. Instead, it is possible that persistent exposures are responsible for the gradual dysfunction that manifests across myriad cellular pathways throughout the progression of the disease.

With the evolution of the “exposome” concept, exposomic studies capture the totality of exposures over a complete lifetime, including exogenous and endogenous exposures, and downstream endogenous products along the exposure-disease continuum. Seminal research has postulated that the cause of PD might begin in the gut (Braak et al., 2006). Strong evidence supports that gastrointestinal (GI) systems are gateways to the environment (Klingelhoefer and Reichmann, 2015). Toxins, nutrients, microbiota, and metabolites can interact with the GI tract, initiate the enteric nervous system (ENS), spread into the central nervous system (CNS), and exert remote effects via the gut–brain axis. Moreover, the substances can also be absorbed directly from the GI tract into the circulatory system and influence CNS functions. These factors may subsequently induce long-lasting neuroplastic changes in the host’s gut or brain. As a result, PD pathogenesis studies have focused on the gut environment.

Many studies on the gut environment have revealed microbial imbalance (dysbiosis) in the gut of the patients with PD (Kataoka, 2016; van Kessel and El Aidy, 2019; Nishiwaki et al., 2020; Sampson, 2020; Shen et al., 2021). Compositional alterations in the gut microbiota of patients with PD have been robustly demonstrated. The results of a meta-analysis showed significantly lower abundance levels of *Prevotellaceae*, *Faecalibacterium*, and *Lachnospiraceae* in patients, and significantly higher abundance levels of *Bifidobacteriaceae*, *Ruminococcaceae*, *Verrucomicrobiaceae*, and *Christensenellaceae* in patients with PD (Shen et al., 2021). The meta-analysis of Nishiwaki et al. (2020) reported that the genera *Akkermansia* and *Catabacter*, as well as the members of the family *Akkermansiaceae*, were increased, whereas the genera *Roseburia*, *Faecalibacterium*, and *Lachnospiraceae* ND3007 group were decreased in PD. Romano et al. (2021) reported enrichment of the genera *Lactobacillus*, *Akkermansia*, and *Bifidobacterium* and depletion of bacteria belonging to the *Lachnospiraceae* family and the *Faecalibacterium* genus in PD gut microbiome by meta-analysis. Nevertheless, the functional relevance of alterations in the gut microbiota remains unclear, and a consensus has not been reached on whether a specific microbial signature exists for PD itself.

Actually, the gut microbiota consists of trillions of microbes that can produce a variety of bioactive metabolites that are derived from the microbial fermentation of dietary and nutritional components. The metabolites represent the downstream readout of biological activities that relate to phenotypes (van Kessel and El Aidy, 2019). With the development of mass spectrometry (MS) or nuclear magnetic resonance (NMR) metabonomics, specific metabolites associated with disease phenotypes can be identified based on fecal, plasma, urine, or other biological fluids to establish associations between exposures, biological response, and health effects. Compared with these biological samples, fecal samples contain a wide array of molecules that reflect the integrative gut environment of nutrient ingestion, digestion, absorption, and metabolism by both gut microbiota and the host GI tract. Furthermore, the immediate vicinity of feces with the ENS is a benefit for metabolic products of the host and microbiota to initiate disease progression in the site. Therefore, the fecal metabolome could reflect gut metabolite exposures appropriately and provide new clinico-biological insights into the etiology of PD.

Currently, various factors create challenges for the identification of gut metabolite exposures that show the potential to increase the risk of developing PD. First, there is a huge number of gut metabolites in feces, but relatively little is known about the metabolites. Heretofore, the studies of the gut metabolome have scarcely been investigated in patients with PD (Unger et al., 2016; Vascellari et al., 2020; Tan et al., 2021; Yan et al., 2021). Second, the studies of gut metabolome provide a “snapshot” of the metabolite profile. Although these previous studies have shown significant differences in the levels of gut microbial metabolites compared to the non-PD controls, there are occasionally inconsistent outcomes. The “snapshot” of the metabolite profile is impacted by small sample size, heterogeneous patient populations, variations in methodology, and different statistical methods used for analyses, which could lead to the different outcomes, even though temporal stability of the gut microbiome in patients with PD had been demonstrated (Aho et al., 2019). Third, little is known about which metabolites contribute to increasing the risk of developing PD. The studies regarding the association between the metabolite exposures and the risks of the disease lack the inference of causality, as gut metabolomic profiling could offer the understanding of not only how the metabolites affect the disease, but also how the metabolites are affected by the disease. Therefore, the role of gut metabolite exposures in PD is critically understudied.

Comparative Toxicogenomics Database (CTD) is a robust, publicly available database that aims to advance the understanding of how environmental exposures affect human health (Davis et al., 2021). It focuses on environmental chemicals and provides unique curated data that enable the development of novel hypotheses about the relationships between chemical exposures and diseases (Grondin et al., 2016). A direct chemical-gene statement can be combined with a direct gene-disease statement to generate a chemical–disease inference *via* the shared genes. Indeed, metabolites could be defined as one of the environmental chemicals that influence susceptibility to chronic diseases by affecting the genes (Dunn et al., 2019). This proof-of-concept study is to identify gut metabolites in feces, as environmental exposure risk factors, that are associated with PD and potentially increase the risk for PD by using known toxicology results. Here, we set out to collect the data of gut metabolites whose levels were significantly changed in the fecal samples of patients with PD from the original clinical studies searched from the academic databases, search each candidate metabolite interacting PD gene set from the CTD, identify and validate gut metabolites associated with PD, determine validated gut metabolite impacting on specific biological functions and cellular pathways involved in PD, and uncover etiologic mechanisms linking gut environmental exposures with PD.

## Materials and methods

### Data collection of candidate metabolites

The gut metabolites for identification were collected from the clinical studies on the fecal metabolome of patients with PD. A systematic search of the corresponding studies was conducted in several databases, including EBM Reviews (Ovid), PubMed, Embase (Ovid), MEDLINE (Ovid), and Elsevier ClinicalKey. The Boolean search term was [(bacterial metabolites) OR (microbial metabolites) OR (fecal metabolites) OR (gut metabolites)] AND (Parkinson disease)] in the titles, abstracts, and keywords. Studies were not limited by language and year of publication. The last search was performed on 21 October 2021. We then manually checked the reference lists of relevant reviews and individual studies to identify additional studies that may have been missed.

After the systematic search, literature triage was conducted according to the inclusion and exclusion criteria. The inclusion criteria were as follows: (1) types of studies: a clinical observational study, especially a case-control study; (2) participants: patients with PD diagnosed according to the clinical diagnostic criteria and healthy controls without PD or free of neurological disorders; (3) comparisons: patients with PD and the healthy controls; (4) analytical methods of metabolome: NMR spectroscopy, liquid chromatography (LC)-MS, gas chromatography (GC), and GC-MS; (5) types of metabolomics: untargeted and targeted; (6) samples: feces; and (7) primary outcomes: fecal metabolites. The exclusion criteria were as follows: (1) participants’ antibiotic use within the preceding 3 months or probiotic use within the preceding 1 month; (2) analytical methods of metabolome: computational models; and (3) no data were reported. The selection process was carried out by two authors independently.

The information of eligible studies was extracted. The fecal metabolites whose levels were significantly altered in patients with PD were collected from the eligible studies. According to the types of metabolomics, metabolites collected from the studies of untargeted metabolomics were used for identification, and metabolites collected from the studies of targeted metabolomics were used for validation. Since untargeted metabolomics involves a comprehensive and systematic analysis of metabolites, it is a kind of unbiased analysis that can discover new biomarkers as broadly as possible. Targeted metabolomics involves the analysis of a specific class of metabolites. Untargeted and targeted metabolomics analyses are often used in combination for the discovery and accurate quantification of differential metabolites.

### Application of the comparative toxicogenomics database

Comparative Toxicogenomics Database provides links to the curated and integrating data interactions between chemicals and disease. The candidate metabolites, as environmental chemicals, and their data were searched for identification by using the CTD^1^. A list of chemicals associated with the term “Parkinson’s Disease” in the CTD was searched through curated association. After the names of candidate metabolites were unitized as the CTD special vocabularies, we compared these metabolites to the list of chemicals associated with the term “Parkinson’s Disease” by using the CTD MyVenn tool.

The human genome background, human genes that were associated with PD, the metabolite-interacting genes, and the metabolite-interacting genes that were associated with PD were searched in the CTD by the Gene Query. The metabolites were dropped for further analysis if “No gene matched your query” was shown in CTD online queries. All data were searched for enrichment analyses, cross-validation, and proportional reporting ratios (PRRs).

### Statistical tests

#### Identification by enrichment analyses

To identify the metabolites that were associated with the PD genes from the candidate metabolites that were collected from the studies of untargeted metabolomics, enrichment analyses were conducted using CTD data as recommended by a previous study (Harris et al., 2020). When the proportion of the metabolite-interacting PD gene set in the PD genes was significantly more than random chance compared to that of the metabolite-interacting human gene set in the human background, the metabolite was identified and its interacting gene set was enriched in genes annotated to the CTD disease term “Parkinson’s Disease.” Fisher’s exact test was adopted for measure that metabolite-interacting gene set was enriched for PD genes. *P*-values were computed by probabilities *p* over defined sets of tables (Prob = Σ_*A*_*p*). For 2 × 2 descriptive tables, *a* was the number of the candidate metabolite-interacting gene set that was overlapped with PD genes; *a+b* was the number of the metabolite-interacting human gene set; *a+c* was the number of human gene sets that were overlapped with the PD genes; and *a+b+c+d* was the number of gene sets in the human background. According to the searched data, data of the cells in 2 × 2 descriptive tables (a, b, c, and d) were calculated and obtained. In all cases of multiple comparisons, Benjamini–Hochberg false discovery rate (FDR) correction was used, with a significance threshold of FDR-corrected *p* < 0.05. The analyses were conducted in *R* statistics software (version 3.1.0) and the Statistical Package for Social Sciences (SPSS, version 19; IBM, Armonk, NY, United States).

#### Confirmation by cross-validation

To confirm the identified metabolites associated with PD genes and avoid false-positive results, a 10-fold cross-validation was performed for enrichment analyses with the metabolite-targeting PD gene sets. The 45,134 human genes identified in the CTD were divided into 10 subsets randomly. The specific size of each subset reflected the range of genes associated with the metabolites in the subset. The data of each subset was used as a verification set and set aside at a time, and the data of the remaining nine subsets were used as a training set to evaluate the enrichment analyses with the exact PD genes and build a model. This procedure was repeated 10 times, with each metabolite being held out exactly once. If any metabolite-interacting human gene sets in the training set had no gene overlapping with the PD genes, this metabolite was removed. Then, the final average accuracy of the 10 models with each metabolite was used to measure the accuracy of the models. A significance threshold of the average FDR-corrected *p*-value was set at 0.05.

#### Proportional reporting ratios

The PRRs were calculated to verify the magnitude of enrichment for all metabolites showing significant overlapping PD genes than expected by chance. They were calculated from the 2 × 2 tables and identified by the calculation of relative risk (RR) from the formula (a/a+c)/(b/b+d) (Waller et al., 2004). A PRR > 1 indicates enrichment. It was important to understand that when used in this context, they were not meant to actually estimate RR but to assist in efficiently identifying the chemical that relates to disease risk.

### Validation of the identified metabolites

To further validate the *prior* selected metabolites that were associated with PD, metabolites whose levels were significantly altered in the feces of patients with PD were collected from the studies of targeted metabolomics. The raw data of the metabolites in the TXT format were checked online in Venny software (version 2.1) to detect the intersection of metabolites between the selected metabolites and the metabolites reported in the targeted metabolomics studies.

### Application of protein analysis through evolutionary relationships

To gain insight into the specific function of PD that may be influenced by the validated metabolites, we identified disease-specific biological process gene ontology (GO) terms and pathways for the metabolites using PANTHER tools.

First, we used the PANTHER tool to identify enriched GO terms represented by the 106 genes contained in the CTD term “Parkinson’s Disease” due to the stability and sorting hierarchical relations by the PANTHER system (Gene Ontology Consortium, 2021). The PD gene set was directly inputted for GO enrichment analyses in the gene ontology consortium (GOC) website^2^ that is connected to the PANTHER analysis tool. The options of “*Homo sapiens*” and “biological process” were chosen. The GO terms of the FDR < 0.05 were displayed. Second, the main biological processes were selected to exclude redundancy according to the FDR values and hierarchical relations between enriched functional classes. The top five GO terms with the minimum FDR values were chosen as the main biological processes of PD according to the results of enrichment analyses, because the closer the FDR values to zero, the more significant the particular GO terms associated with the PD gene set. Notably, based on hierarchical relations, when the FDR values of two terms were quite close and one term was a child node of the other, we chose the parent term. Then, enriched GO terms represented by the 106 PD genes were identified.

To explore the biological processes and pathways for each validated metabolite, GO analysis and pathway enrichment analysis were performed using PANTHER tools directly from the GOC website. The PANTHER tool was repeatedly applied to identify enriched GO terms among each validated metabolite-interacting human gene set. When the corrected FDR value was below a significance threshold (*p* < 0.05), the GO term was displayed. Further, the GO terms of validated metabolites were identified, if the biological processes enriched in validated metabolite gene sets overlapped with our identified GO terms in PD.

Pathway enrichment analyses were performed using the “PANTHER pathway” tool (Mi and Thomas, 2009) to identify the biological pathways affected by these metabolites, because the “PANTHER pathway” tool focused on both signaling pathways and metabolic pathways. The validated metabolite-interacting human gene sets were inputted for pathway enrichment analyses. The options of “*Homo sapiens*” and “PANTHER pathway” were chosen to “launch.” The pathway terms with an FDR < 0.05 were displayed.

### Visualized networks

According to the targeting PD gene sets of the validated metabolites, the protein–protein interaction (PPI) networks were constructed to dig out metabolite-associated hub genes in PD. The STRING database^3^ was applied for the validated metabolite targeting PD gene sets to construct the PPI networks. The restricted organism was “*Homo sapiens*,” with a confidence score >0.4. Network analyses were conducted by using a Cytoscape (version 3.8.2, San Diego, CA, United States) plug-in CytoHubba, which ranked the top 10 genes in the networks by Maximal Clique Centrality (MCC) scores and visualized, ultimately to uncover metabolite-associated hub genes in the PPI network (Shannon et al., 2003).

## Results

The literature collection was performed up to 21 October 2021. After literature triage and selection, four unique studies were eligible. The key characteristics of included studies are displayed in Table 1.

**TABLE 1:** Characteristics of eligible studies.

Author, year	Study design	Location (City, Country)	Participants and interventions	Measurement	Type of metabonomics	Presentation of results
Tan et al., 2021	Clinical observational study	Kuala Lumpur, Malaysia	Patients with PD (*n* = 104) their spouse (*n* = 91) or sibling (*n* = 5) controls living in the same community. For PD patients: anti-parkinsonian medication initiation within the preceding 3 months or adjustment within the preceding month.	NMR spectroscopy and LC-MS	Untargeted	**Increased:** Long-chain saturated fatty acids (17-octadecynoic-acid and *cis*-9, 10-epoxystearic acid), sphingolipids (ceramide [Cer(d14:1(4E)/22:0(2OH)] and dehydrophytosphingosine), and glycolysis products (ethanol and scyllo-inositol); **Decreased:** Ubiquinones (coenzymes Q6 and Q9), ceramide (Cer(d18:0/14:0)), butyrate, amino acids (glutamate and tyrosine), and substrates of choline metabolism [choline, phosphocholine, trimethylamine, trimethylamine N-oxide (TMAO)] and energy metabolism (pyruvate and fumarate).
Vascellari et al., 2020	Clinical observational study	Cagliari, Italy	64 patients with diagnosed idiopathic PD and 51 healthy controls, selected among spouses and family members of study patients. Patients were received stable doses of dopaminergic treatment for at least 4 weeks before enrollment.	GC-MS	Untargeted	**Increased:** Cadaverine, ethanolamine, hydroxypropionic acid, isoleucine, leucine, phenylalanine, and thymine; **Decreased:** Linoleic acid, oleic acid, nicotinic acid, glutamic acid, pantothenic acid, pyroglutamic acid, succinic acid, and sebacic acid.
Unger et al., 2016	Clinical observational study	Homburg, Germany	34 PD patients and 34 age-matched controls. All 34 PD patients were on dopaminergic drugs	GC	Targeted	**Decreased:** Acetate, propionate and butyrate.
Yan et al., 2021	Clinical observational study	Harbin, China	20 PD patients and 20 age, body mass index and gender-matched healthy controls. All PD patients were using anti-parkinsonian medications	GC-MS	Targeted	**Decreased:** Isoleucine, valine, phenylalanine, tyrosine and tryptophan.

GC-MS, Gas chromatography-mass spectrometry; NMR, nuclear magnetic resonance; LC-MS, liquid chromatography-mass spectrometry; SCFA, Short chain fatty acids; PD, Parkinson’s disease.

### Identification and validation of metabolites that were associated with Parkinson’s disease

In the four studies, 33 of the 36 metabolites whose names matched the CTD special vocabularies were chosen, and the other three metabolites were removed. In the CTD, 187 chemicals linked to the term “Parkinson’s Disease” were identified through curated association via genes, whereas none of these 33 candidate metabolites overlapped with the 187 chemicals (data are not shown).

**FIGURE 1:**
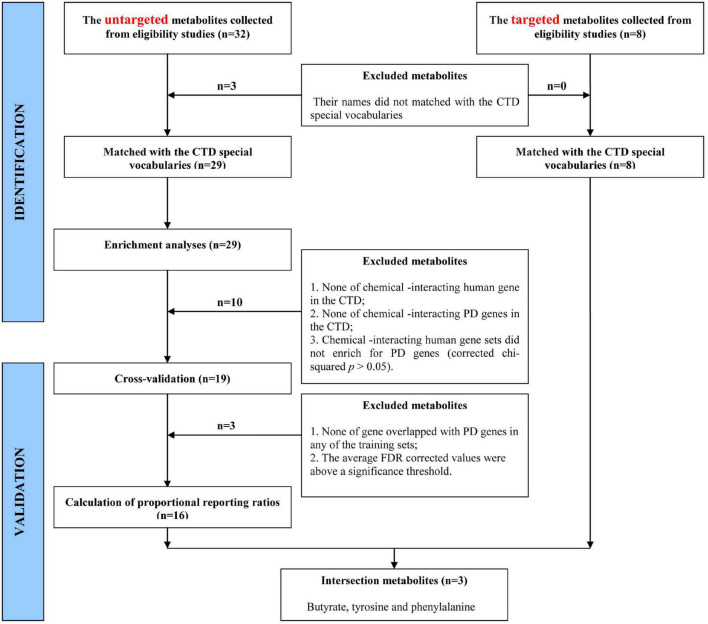
The flow diagram showing metabolite identification and validation processes.

Then, we identified and validated metabolites that were associated with PD. The identification and validation processes of metabolites are shown in the flow diagram (Figure 1 and Supplementary Table 1).

First, we identified metabolites that were associated with PD genes by using the CTD curated database through chemical–gene interactions and gene–disease relationships to discover chemical–disease connections. Among the eligible studies, Vascellari et al. (2020) and Tan et al. (2021) explored the untargeted metabolome in feces. In the two studies, 32 candidate metabolites whose levels were significantly changed in the feces of PD patients were detected. Further, 29 of the 32 metabolites whose names matched the CTD special vocabularies were chosen. The enrichment analyses required that the data were collected from the CTD. The human genome background (45,134 human genes in total) and 106 human genes linked to the clinical term for “Parkinson’s Disease” were searched and downloaded. We surveyed 29 metabolites for their associated human genes and targeted PD genes in the CTD. Four out of the 29 metabolites could not be tested for enrichment due to the absence of a chemical-interacting gene in the CTD, including 17-octadecynoic acid, 9, 10-epoxystearic acid, ubiquinone 6, and scyllitol. Six out of the 25 metabolites, including isoleucine, ethanolamine, succinic acid, trimethyloxamine, ubiquinone 9, and trimethylamine, were also dropped because the metabolites were not associated with any PD gene in the CTD curated database. The enrichment analyses showed that 19 metabolites were enriched for PD genes (corrected chi-squared *p* < 0.05).

Second, the results of cross-validation of enrichment analyses showed that four out of the 19 metabolites, including sebacic acid, cadaverine, thymine, and tryptophan, were dropped, because at least one gene set of metabolites in their training sets had none of the genes overlapped with the targeting PD genes, or their average FDR-corrected values were above a significance threshold. Finally, the 16 metabolites that were associated with PD were confirmed.

Third, we observed the PRR values of prioritized metabolites were more than 1 (i.e., PRRs > 1), which verified these metabolites were enriched for PD genes and reflected that they were associated with a higher risk of PD due to more overlap with the PD genes. The highest PRRs were observed for pyroglutamic acid (77.3), tyrosine (75.0), pyruvate (47.2), linoleic acid (42.3), and phenylalanine (36.1) (Supplementary Table 1).

**FIGURE 2:**
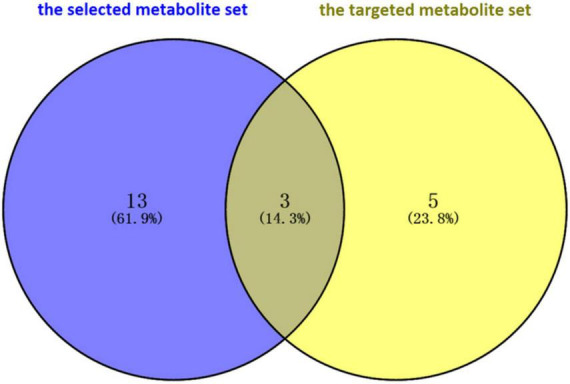
Validation of intersection metabolites in the selected metabolite set and the targeted metabolite set through Venn diagram software. A total of three intersection metabolites, including butyrate, tyrosine, and phenylalanine, were obtained. Then, the three metabolites (butyrate, tyrosine, and phenylalanine) were validated that they were highly associated with Parkinson’s disease.

Finally, the *prior* selected metabolites associated with PD were validated through comparison with metabolites whose levels were significantly altered in the feces of PD patients, and these were collected from the studies of targeted metabolomics. Unger et al. (2016) and Yan et al. (2021) determined the concentration of short-chain fatty acids (SCFAs) and amino acids, respectively, by using the targeted metabolomics technique. Eight metabolites were detected in the two studies. A total of three intersection metabolites, including butyrate, tyrosine, and phenylalanine, were obtained between the selected metabolites and the metabolites reported in the targeted metabolome studies by using the Venn diagram (Figure 2). Then, the three metabolites (butyrate, tyrosine, and phenylalanine) were validated that they were highly associated with PD.

### Functional analyses of validated metabolite gene sets

**FIGURE 3:**
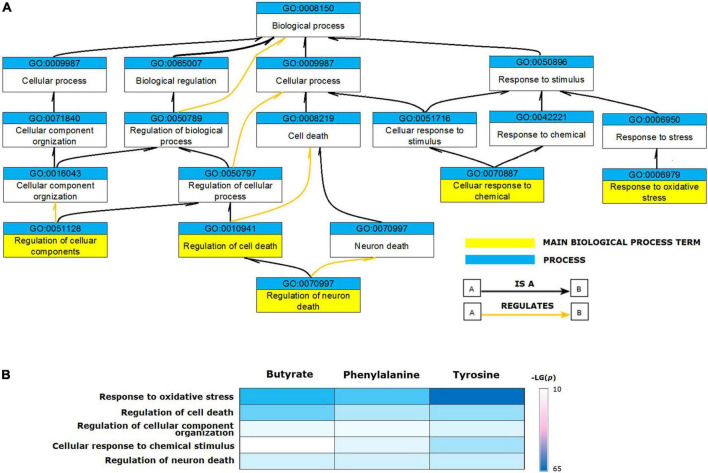
Biological processes enriched for validated metabolite gene sets. **(A)** The ancestor chart for enriched GO terms represented by the 106 genes contained in the CTD term “Parkinson’s Disease”. **(B)** The validated metabolites influenced five main biological process GO terms associated with Parkinson’s disease.

The validated metabolite gene sets impacting the PD biological processes and cellular pathways were identified. First of all, functional analyses of the PD gene set were performed, since the human genes that had a curated association with PD are likely to be the common core of PD etiology. Enriched GO terms represented by the 106 genes contained in the CTD term “Parkinson’s Disease” were identified. The GO enrichment analyses of the 106 PD gene set identified 1,160 enriched biological process terms (FDR < 0.05). After sorting by the FDR values and parent–child term hierarchical relationships, the 106 PD gene set was enriched in five main biological process terms, including “regulation of neuron death” (GO: 1901214, FDR = 7.88E-23), “regulation of cell death” (GO: 0010941, FDR = 5.55E-19), “response to oxidative stress” (GO: 0006979, FDR = 1.81E-15), “cellular response to chemical stimulus” (GO: 0070887, FDR = 2.12E-15), and “regulation of cellular component organization” (GO: 0051128, FDR = 2.56E-15) (Figure 3A).

Based on these results, we identified the enriched GO terms for functional analysis of validated metabolites. The relevant processes enriched for PD genes were also enriched for the validated metabolite gene sets (Figure 3B). All validated metabolites influenced the GO terms of “response to oxidative stress,” “regulation of cell death,” “regulation of neuron death,” “cellular response to chemical stimulus,” and “regulation of cellular component organization.”

**FIGURE 4:**
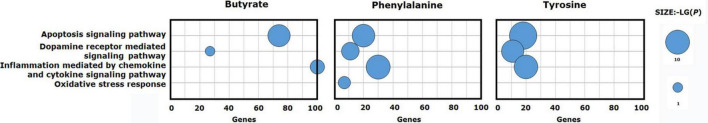
Signaling pathways enriched for validated metabolite gene sets. Phenylalanine impacting gene set was enriched for the four main pathways. Butyrate and tyrosine impacting gene sets were enriched for three of the four main pathways.

To understand the possible pathway of these three validated metabolites, pathway enrichment was re-analyzed via the PANTHER pathway tool. Based on the current understanding of the etiology of PD, pathway analysis identified four distinct pathways that were involved in the “apoptosis signaling pathway,” “dopamine receptor mediated signaling pathway,” “inflammation mediated by chemokine and cytokine signaling pathway,” and “oxidative stress response.” Phenylalanine impacting gene set was enriched in the four main pathways. Butyrate and tyrosine impacting gene sets were enriched for “apoptosis signaling pathway,” “dopamine receptor mediated signaling pathway,” and “inflammation mediated by chemokine and cytokine signaling pathway” (Figure 4).

### Identification of metabolite-associated hub genes

**FIGURE 5:**
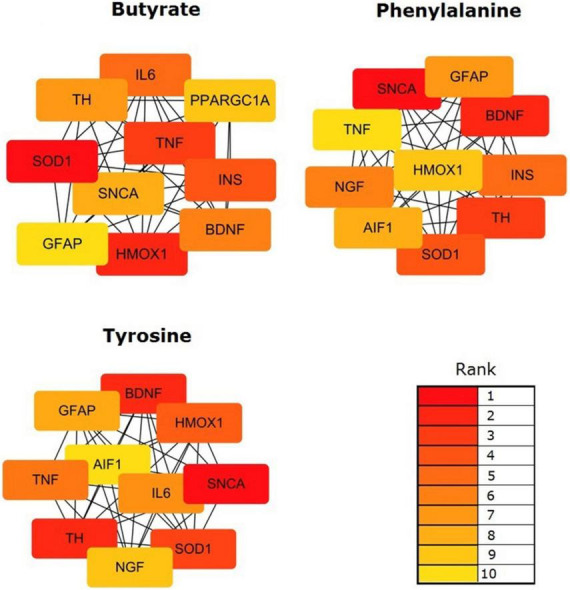
Identification of the hub genes among the targeting PD gene sets of the validated metabolites. Network analyses were conducted by using a Cytoscape plug-in CytoHubba. The top 10 genes in the networks were identified by Maximal Clique Centrality (MCC) scores and visualized.

The topological results of the visualized network identified the hub genes in the targeting PD gene sets of the validated metabolites. The top 10 hub genes impacted by validated metabolites are shown in Figure 5. Butyrate targeting PD hub genes were SOD1, HMOX1, TNF, INS, IL6, BDNF, TH, SNCA, PPARGC1A, and GFAP; tyrosine targeting PD hub genes were SNCA, TH, BDNF, SOD1, HMOX1, TNF, IL6, GFAP, NGF, and AIF1; and phenylalanine targeting PD hub genes were SNCA, BDNF, TH, SOD1, INS, NGF, GFAP, AIF1, HMOX1, and TNF. The 12 hub genes were impacted by at least one of the three validated metabolites. Six of them were impacted by all validated metabolites, including alpha-synuclein (SNCA), tyrosine 3-monooxygenase (TH), brain-derived neurotrophic factor (BDNF), superoxide dismutase 1 (SOD1), heme oxygenase-1 (HMOX1), tumor necrosis factor (TNF), and glial fibrillary acidic protein (GFAP).

## Discussion

Environmental exposures are known to be the risk factors for PD; however, gut environmental risk factors for PD are critically understudied. The present study identifies and validates gut metabolites in feces in feces, as environmental exposure risk factors, that were associated with PD and potentially increase the risk for PD. After collection of the candidate metabolites from the eligible literature, a set of gut metabolites whose levels are significantly changed in the feces of PD patients are collected from the studies of untargeted metabolomics to evaluate whether the metabolites are associated with the PD genes based on the publicly available toxicogenomic data. Sixteen metabolites are identified and divided into the main categories according to their structures and biological functions: alcohols (ethanol), amino acids (leucine, phenylalanine, pyroglutamic acid, glutamate, and tyrosine), short-chain fatty acids (SCFAs, propionate, and butyrate), unsaturated fatty acids (linoleic acid and oleic acid), energy metabolism (lactate, pyruvate, and fumarate), vitamins (nicotinic acid and pantothenic acid), and choline metabolism (choline). Finally, a total of three identified metabolites, including butyrate, tyrosine, and phenylalanine, were validated that were associated with PD through comparison with the targeted metabolites whose levels were significantly altered in the feces of PD patients.

The candidate gut metabolites were collected from the eligible literature according to the inclusion and exclusion criteria. These original data of clinical studies were robust to permutation testing. Given the diversity in gut metabolite structures that were enriched with PD genes, the diversity in gut metabolite sources from gut microbiota or host, and the diversity of gut metabolites known or suspected to cause PD, it seems unlikely that a generalizable set of characteristics will identify gut metabolites that present a hazard for this endpoint. Our study leverage known toxicology results to identify the gut metabolites associated with PD. Then, the results of the study contribute to the body of evidence that exposure to butyrate, tyrosine, and phenylalanine constitutes risk factors for PD.

Increased gut permeability and inflammation in patients with PD have been hypothesized to be linked to low gastrointestinal SCFAs. However, there is no direct evidence that SCFAs were correlated with PD or have a known or potential therapeutic role in PD. Our findings indicate that SCFAs, particularly butyrate, affect more PD genes than expected by chance. It has been proven that exposure to specific SCFAs, especially butyrate in the host body, seems to influence the development of PD. SCFAs are the primary end products of fermentation of non-digestible carbohydrates (NDCs) produced by the colonic microbiota. For example, butyrate is produced by *Clostridium tyrobutyricum*, while acetate and propionate are produced by *Bacteroides thetaiotaomicron* (Baldini et al., 2020). The alteration in the SCFA-producing bacteria has emerged as the most consistent gut microbiome alteration associated with PD (Romano et al., 2021). Naturally, a reduction in SCFAs was observed in the fecal samples of PD patients, in a manner consistent with the observed changes in the gut microbiota composition (Unger et al., 2016; Tan et al., 2021). Their role in PD pathogenesis is to act locally on the colonic mucosa and exert remote effects via the ENS. Butyrate increases the proportion of choline acetyltransferase-immunoreactive neurons to regulate colonic mucosal homeostasis, inhibits activation of NF-κB and degradation of IκBα to reduce the expression of pro-inflammatory cytokines to regulate mucosal immune response, and increases colonic motility in rats (Sun and Shen, 2018).

Our analyses of fecal metabolites identify and validate that several amino acids, including tyrosine and phenylalanine, affect more PD genes than expected by chance. It is determined that exposure to tyrosine and phenylalanine is associated with PD. Tyrosine is a precursor to catecholamine neurotransmitters, including serotonin, DA and norepinephrine, the synthesis and release of which are sensitive to relatively small, physiological changes in precursor concentrations. DA is one of the neurotransmitters that play a critical role in motor control, and increasing DA function in the brain improves PD symptoms. Phenylalanine, being the precursor to tyrosine, is involved in the syntheses of neurotransmitters and hormones, and the body’s lipid and carbohydrate metabolisms. As such, tyrosine and phenylalanine could contribute to the pathogenesis of PD. Of note, Tan et al. (2021) and Yan et al. (2021) found the same outcomes that fecal levels of tyrosine were significantly reduced in patients with PD compared to controls, while the discrepant outcomes in the fecal phenylalanine levels in patients with PD were reported (Vascellari et al., 2020; Yan et al., 2021). Generally, except for a few amino acids produced from the *de novo* synthesis of the gut microbiota, most of the amino acids in the gut originate from the metabolism of host dietary proteins and tissue proteins or the transformation of other nitrogenous substances. Actually; phenylalanine is an essential amino acid, and tyrosine is synthesized by the hydroxylation of phenylalanine. Tyrosine becomes an essential amino acid when there is a lack of phenylalanine, hence their concentrations in the fecal samples are primarily dependent on the host’s dietary composition. Moreover, tyrosine and phenylalanine belong to aromatic amino acids, as well as the large neutral amino acids (LNAA). Aromatic amino acids (tryptophan, phenylalanine, tyrosine, and also L-dopa) are transported into the brain or the intestinal cells via LNAA transporters (Fernstrom, 2013; Lu, 2019; Shao et al., 2021). The transporters are competitive and saturable, so raising the dose of one LNAA increases the intestinal absorption of that LNAA, and reduces the absorption of others. Obviously, the intestinal absorption of essential amino acids can have an impact on the concentrations of these amino acids in the fecal samples. In the two abovementioned studies, Vascellari et al. (2020) included all patients with PD who received stable doses of L-dopa treatment for at least 4 weeks before enrollment, and Yan et al. (2021) reported that PD patients were using anti-parkinsonian medications, but the details were not described. Maybe, the difference in the drug therapy is an underlying reason for the conflicting outcomes, which implies that L-dopa treatment may affect the metabolism of aromatic amino acids, particularly phenylalanine, in patients with PD.

In our results, some of the identified gut metabolites affected more PD genes than occurred by chance, but were not validated. They are not necessarily irrelevant to PD, but rather the hitherto studies regarding targeted metabolomics did not cover all metabolites in human feces, including energy metabolism (lactate, pyruvate, and fumarate), unsaturated fatty acids (linoleic acid and oleic acid), vitamins (nicotinic acid and pantothenic acid), choline metabolism (choline), and alcohols (ethanol). Though these metabolites are mostly simple and non-specific molecules, exposure to these gut metabolites appears to play a role in PD pathogenesis and cannot be ignored.

Among the nine metabolites, lactate, fumarate, and pyruvate were highly enriched for PD genes and involved in energy metabolism. Pyruvate, a key molecule critical for energy metabolism (Gray et al., 2014), is the end product of glycolysis in the cytoplasm and becomes a major substrate for the tricarboxylic acid (TCA) cycle. In the aerobic condition, pyruvate, in mitochondria, generates several organic acids, such as fumarate and succinate through the TCA cycle to produce ATP, NADH, and FADH2, which ultimately provide energy. In the absence or scarce oxygen, glycolytic pyruvate is converted into lactate to provide an energy source through anaerobic glycolysis, thus pyruvate is prevented from undergoing mitochondrial oxidative phosphorylation (OxPHOS). Mitochondrial dysfunction causes high lactate production, because the “aerobic glycolysis” is a metabolic compensation for a reduction of the activity of mitochondrial electron chain transport. Early literature has observed decreased fumarate and pyruvate levels and increased lactate levels in the feces of patients with PD (Ghosh et al., 2016; Quansah et al., 2018). The present results obtained by enrichment analyses and PRRs identify that lactate, fumarate, and pyruvate are associated with PD. It indicates that mitochondrial dysfunction, as an initiating factor (Malpartida et al., 2021), plays an important role in contributing to the development and progression of PD.

Unsaturated fatty acids are important components of nerve cell membranes and have neuroprotective, antioxidant, and anti-inflammatory properties. Linoleic acid is a doubly unsaturated fatty acid and is also known as an essential fatty acid. In the Farming and Exercise Evaluation (FAME) Study, a nested case-control study in the United States, α-linolenic acid (αLNA) intake was found to be inversely correlated with a dose–response trend for PD (Kamel et al., 2014). A Greek cohort of the European Prospective Study into Cancer and Nutrition (EPIC) revealed that aLNA and linoleic acid were significantly associated with a reduced risk of PD (Kyrozis et al., 2013). Moreover, it was reported that the decreased linoleic acid level was positively associated with a reduced abundance of *Bacteroidaceae* in the stool samples of PD patients (Vascellari et al., 2020). Oleic acid is an unsaturated C-18 or an omega-9 fatty acid, which is a component of the normal human diet. Li et al. (2019) revealed that oleic acid decreased the death process by inhibiting the production of excessive reactive oxygen species (ROS) and fatty acids, thereby protecting the mitochondria in a PD model. Thus, the protective effects of linoleic acid and oleic acid on PD are confirmed. Patients with PD are characterized by reduced linoleic acid and oleic acid levels in the fecal samples, which are associated with an increased risk of PD.

Nicotinic acid is a water-soluble vitamin and is derived from feed supplementation, tryptophan metabolism, and microbial synthesis, such as *Lachnospira, Pseudobutyrivibrio*, and *Roseburia* genera (Vascellari et al., 2020). It displays anti-inflammatory, antioxidant, and protective effects against neurodegenerative mechanisms. Some of the PD symptoms, such as sleep dysfunction, fatigue, and mood changes, appear to be consistent with nicotinic acid deficiency. Enhancement of the nicotinic acid levels has the potential to maintain or improve the quality of life and slow disease progression in patients with PD (Chong et al., 2021). Pantothenic acid is also a water-soluble vitamin that is required for coenzyme A (CoA) synthesis. Pantothenic acid acts on normal epithelial organs, such as nerves, glands, adrenal, skin, and digestive tract *in vivo*, improving the resistance of animals to pathogens. Scholefield et al. (2021) reported that pantothenic acid was significantly decreased in the cerebellum, substantia nigra (SN), and medulla in dementia patients with PD. Our enrichment analytical results indicate that the decreased concentrations of nicotinic acid and pantothenic acid in feces could translate into functional losses, thus affecting the disease phenotype.

It is presented that metabolites involved in choline metabolism, especially choline, exhibit enrichment for PD genes. Choline is an essential nutrient predominantly obtained from dietary supplementation. It plays a role in the synthesis of essential lipid components of the cell membranes, phosphatidylcholine by intermediate phosphocholine, and in the synthesis of the neurotransmitter acetylcholine by the enzyme choline acetyltransferase. Comparative analyses indicated abnormality in the choline metabolism in neurodegenerative diseases. Tan et al. (2021) observed that choline levels were decreased in the fecal samples of PD. Indeed, the deregulation of choline metabolic pathways has profound effects on cellular physiology in PD. On one hand, the decreased choline levels may affect the synthesis of acetylcholine and regulation of colonic motility, ultimately leading to GI symptoms, such as constipation and defecatory dysfunctions. On the other hand, choline deficiency induces p53 independent apoptosis that is associated with TGFb1 signaling and ROS production, thus disrupting membrane potentials and resulting in mitochondrial dysfunction and cell death (Michel et al., 2006). Dietary choline supplementation improves a variety of physical functions, including cognition, learning, and memory. Our findings again highlight abnormal choline metabolism in PD.

Ethanol is a prioritized metabolite that targets more PD genes than random chance by the identification of enrichment analyses. Small amounts of ethanol are endogenously produced by gut microbes through anaerobic fermentation, while most of the ethanol detected in biological fluids and tissues is likely from alcohol consumption. Alcohol consumption is one of the environmental factors contributing to PD (Noyce et al., 2012). However, the relationship between alcohol consumption and PD is complex. First, ethanol in the body is converted to acetaldehyde which is a highly active and toxic compound in the ethanol metabolic pathway that enhances 1-methyl-4-phenyl tetrahydropyridine (MPTP)-induced parkinsonism in mice (Peng et al., 2020). Second, alcohol intake alters the microbiota and the microbiota–gut–brain axis. Alcohol-induced microbiome changes can enhance gut permeability and neuroinflammation, and affect the balance of the neuroimmune function (Bode and Bode, 2003). Specifically, increased gut permeability due to alcohol abuse leads to elevated levels of lipopolysaccharide (LPS) in the portal blood flow that binds to TLR4 and activates NF-κB, subsequently stimulating pro-inflammatory cytokine release, ROS production, and oxidative stress (Meroni et al., 2019). Therefore, ethanol targeting more PD genes than random chance has been identified, which indicates that a relationship exists between ethanol exposure and the pathogenesis of PD.

The PD genes were enriched in the GO biological processes, including “regulation of neuron death,” “regulation of cell death,” “response to oxidative stress,” “cellular response to chemical stimulus,” and “regulation of cellular component organization.” These main biological processes are identified as phenotypes associated with PD in the CTD and are consistent with the known etiologies of PD. The five main biological processes were also enriched for all three validated gut metabolites. Then, the identified main biological processes are integrated to elaborate an association between environmental exposures and PD pathogenesis. The mitochondria are the major sites of ROS production, and are particularly susceptible to oxidative stress-induced damage. During mitochondrial dysfunction, oxidative stress becomes a key driver of the complex degenerating cascade underlying dopaminergic neurodegeneration (Trist et al., 2019), resulting in the loss of dopaminergic neurons in the SNpc and ultimately leading to motor dysfunction. Notably, the validated gut metabolites, including butyrate, tyrosine, and phenylalanine, may be involved in PD pathogenesis through “cellular response to chemical stimulus.” Hence, it is considered that gut metabolite exposure might trigger and maintain the pathogenesis of PD.

Furthermore, it was found that PD genes were enriched in a series of different signaling pathways, such as “apoptosis signaling pathway,” “dopamine receptor mediated signaling pathway,” “inflammation mediated by chemokine and cytokine signaling pathway,” and “oxidative stress response.” Apoptosis, the major pathway for programmed cell death (PCD), has been implicated as the main mechanism of neuronal death and DA deficiency in PD. Neuroinflammation is marked by the activation of microglia and reactive astrocytes in the brain parenchyma, as well as in the release of various inflammatory mediators, including cytokines (Lin et al., 2019) and chemokines. Indeed, cytokines can promote the apoptosis of neurons, oligodendrocytes, and astrocytes and cause damage to the myelinated axons. However, they also exhibit neuroprotective effects independent of their immunomodulatory properties (Policastro et al., 2020), which are involved in immunological responses, and play an important role in the development and progression of PD (Hirsch and Hunot, 2009; Chu et al., 2012). Meanwhile, chemokines act mainly as mediators of leukocyte recruitment to inflammatory sites (Scalzo et al., 2011) and modulate neurotransmitter release that is regulated by neuronal excitability and play a key role in the pathogenesis of PD (Rostène, 2010). Among the three validated metabolites, phenylalanine was enriched in the four pathways. Butyrate and tyrosine were enriched in the apoptosis signaling pathway, inflammation mediated by chemokine and cytokine signaling pathway, and dopamine receptor mediated signaling pathway. These observations provide a potential mechanistic link between gut metabolite exposure and PD.

Finally, our results presented that 12 PD hub genes were influenced by at least one of the three validated metabolites. Six of them, SNCA, TH, BDNF, SOD1, HMOX1, TNF, and GFAP, were impacted by all the validated metabolites. Within the PD hub genes, SNCA, the gene encoding for a-synuclein, is a pivotal PD-associated gene (Kalia and Lang, 2015). It may be involved in the regulation of the misfolding and aggregation of a-synuclein, dopamine release and transport, induce fibrillization of microtubule-associated protein tau, and reduce neuronal responsiveness to various apoptotic stimuli, leading to decreased caspase-3 activation. Other hub genes also influence the ability of the host to synthesize dopamine (TH), inflammatory responses (TNF, IL6, and AIF1) (Alonso-Navarro et al., 2014), oxidative stress (HMOX1 and SOD1) (Cuadrado and Rojo, 2008; Chang and Chen, 2020), neurotrophy and protection (BDNF, NGF, and GFAP), and energy metabolism (INS and PPARGC1A). The gut metabolites provide biological functions *via* regulating host gene expression and are involved in the pathophysiology of PD.

Our findings identify the gut metabolites that are associated with PD. Among these metabolites, most of them are host–microbial co-metabolites found in feces. The findings prove that exposure of the gut to environmental factors from the host diet and metabolism of host and gut microbiota may be potential etiologies of PD. Identification of gut metabolite exposures can provide biomarkers for the identification of the disease, facilitate an understanding of the relationship between gut metabolite exposures and response, and present an opportunity for PD prevention and therapies.

## Limitations of the study

There are a number of limitations to this study. First, some gut metabolites that were identified, but not validated, are not necessarily irrelevant to PD, but rather the hitherto studies regarding targeted metabolomics did not cover all metabolites in human feces. The more targeted metabolomics studies toward all the detected metabolites should be further conducted. Second, the analyses could not address dose–response relationship of metabolites. Our discussions on the association between fecal metabolite levels and increasing PD risk were based on the results of the previous literature. Third, identification of metabolites *via* a number of curated gene interactions, but not all PD-associated genes in humans have been determined, and the metabolite–gene interactions should be elucidated in further studies.

## Data availability statement

The original contributions presented in this study are included in the article/Supplementary material, further inquiries can be directed to the corresponding authors.

## Author contributions

JY: writing of the original draft. XF: reviewing and editing. XZ: data curation and visualization. MZ: formal analysis. HX: data curation, formal analysis, and supervision. RL: reviewing and editing of the manuscript and supervision. HS: conceptualization and methodology. All authors contributed to the article and approved the submitted version.

## Conflict of interest

The authors declare that the research was conducted in the absence of any commercial or financial relationships that could be construed as a potential conflict of interest.

## Publisher’s note

All claims expressed in this article are solely those of the authors and do not necessarily represent those of their affiliated organizations, or those of the publisher, the editors and the reviewers. Any product that may be evaluated in this article, or claim that may be made by its manufacturer, is not guaranteed or endorsed by the publisher.

## Footnotes

1
http://ctdbase.org/

2
http://geneontology.org/

3
http://string-db.org
